# Risk-taking behavior, the second-to-fourth digit ratio and psychological features in a sample of cavers

**DOI:** 10.7717/peerj.8029

**Published:** 2019-11-08

**Authors:** Sergio Rinella, Andrea Buscemi, Simona Massimino, Vincenzo Perciavalle, Marta Maria Tortorici, Daria Ghiunè Tomaselli, Valentina Perciavalle, Donatella Di Corrado, Marinella Coco

**Affiliations:** 1 Department of Biomedical and Biotechnological Sciences, University of Catania, Catania, Italy, Italy; 2 Department of Research, Horus Social Cooperative, Ragusa, Italy, Italy; 3 Department of Sciences of Formation, University of Catania, Catania, Italy, Italy; 4 Department of Sport Sciences, Kore University, Enna, Italy

**Keywords:** High-risk sport, Mood state, Digit ratio, Anxiety, Personality

## Abstract

**Background:**

The risk-taking behavior is largely modulated by the subject’s history, its lifestyles, by the characteristics of the situations with which it is compared, and also by the effects of prenatal androgens. Thus, the personality of the single person is a significant predictor of such way of acting.

**Methods:**

The present study aimed to explore the relationship between Digit Ratio Measurement (2D:4D) and personality factors capable to be good predictors for choosing highly risky activities, such as caving. Furthermore, our purpose was to investigate whether 2D:4D ratio is related to cavers’ affective states and to assess the personological and emotional features of 34 healthy cavers, aged between 24 and 71 years (*M* = 39.70, *SD* = 9.81).

**Results:**

Data analysis showed several significant correlations between 2D:4D and Deliberate Risk Taking (RTI) and Precautionary Behavior (RTI), confirming that 2D:4D is a reliable index able to predict risk-taking behaviors. Furthermore, data analysis showed that Conscientiousness and its sub-dimension *Scrupulousness* (BFQ-2) are recurrent among significant correlations; in particular, the latter reports negative correlations with many factors of POMS. Moreover, all participants seemed to have a good attitude to collaboration, in terms of goal-direct strategy, and an adequate management of negative affective states, useful to maintaining a good level of stress within the group. Finally, the BFQ-2 factor *Openness to culture* seemed to be a predominant feature in the cavers, and this feature could be considered as predictive in the choice of an activity, such as caving, which requires curiosity, perseverance and a great planning of cave exploration.

## Introduction

The risk-taking behavior is largely modulated by the subject’s history, its conditions and lifestyles, and by the characteristics of the situations with which it is compared (Boyer Ty, 2006).

Thus, the personality of the single person is a significant predictor of such way of acting; specifically, we have found in previous studies that two factors of the Big Five Theory (Costa & McCrae, 1990; Digman, 1990), Extraversion and Conscientiousness, are very important to explore risk-taking behaviors (Bermúdez, 1999; Vollrath & Torgersen, 2002; Clarke & Robertson, 2005).

The Extraversion, or Energy, refers to the quality and intensity of interpersonal relationships, the level of activity, the need for stimulation and the ability to experience joy. The Conscientiousness points out the individual’s degree of organization, perseverance and impulse to a goal-directed behavior. It distinguishes secure and demanding subjects from the sloppy and indolent ones. Low Conscientiousness would be negatively related to risk-taking behaviors (Castanier, Le Scanff & Woodman, 2010).

In addition, Barlow, Woodman & Hardy (2013) have confirmed the role of Conscientiousness and Extraversion related to risk-taking domain. In fact, they have found that Conscientiousness has been consistently associated with precautionary behaviors and, conversely, high Extraversion have been associated with deliberate risk-taking.

One of the reasons that conduct people to undertake in high-risk activities could be linked to the individual’s desire to build significant interpersonal relationships (Celsi, Rose & Leigh, 1993).

Precedent studies emphasize that individuals performing stressful and risky activities, would be able to improve the regulation of their anxiety and emotions (Tulloch & Lupton, 2003; Lyng, 2005).

Furthermore, it has been proposed that brain development is influenced by prenatal androgens that would enhance its sensitivity to testosterone during life (Tobet & Baum, 1987; Breedlove & Hampson, 2002). By these conditions may result increased self-confidence (Boissy & Bouissou, 1994), search persistence (Andrew & Rogers, 1972) and risk choice (Booth, Johnson & Granger, 1999; Apicella et al., 2008), as well as strong vigilance and speedy reaction times (Salminen et al., 2004). Some marker would be able to assess the effects of prenatal androgens (Cohen-Bendahan, Van de Beek & Berenbaum, 2005), but the most suited is probably to be the second-to-fourth digit length (2D:4D) ratio. Moreover, this factor seems to be predictive of success among high financial brokers (Coates, Gurnell & Rustichini, 2009), or correlated with success in medical schools of state-run Italian Universities (Coco et al., 2011), linked to performance of competitive sports, such as basketball (Tester & Campbell, 2007), skiing (Manning, 2002) and soccer (Manning & Taylor, 2001; Perciavalle et al., 2013).

In addition, Barlow, Woodman & Hardy (2013) have presented a research to contest the opinion that all high-risk events are identical and focused only by perception seeking, as it has been historically considered (Zuckerman, 1971). In fact, the authors aimed to examine the different motives for two contextually specific high-risk activities, skydiving and mountaineering, using perception seeking, emotion regulation and agency perspectives as measure of motives for behavior. The aim of their research was twofold: to challenge the widely held view that high-risk participants can be considered a homogeneous perception seeking group and to understand the underlying motives for high-risk, long-duration, low-perception activities such as mountaineering. This study demonstrated for the first time that there exist different motives for what has been long considered only a class of controlled risk taking, for example, some risk takers (e.g., skydivers) are moved by the feeling recompenses of their activity, while the agentic emotion regulation processes of their activity motivate others (e.g., mountaineers). This final explanation is mainly informative, as it suggests that risk takers can be motivated by the opportunity of an improved future state through a high expectation of their life. In any case, the principal function of the high-risk field is that individuals expect to feel greater sensation regulation during their high-risk activity. Lastly, the agentic sensation regulation recommends that such benefits be transferred to other essential aspects of life (Woodman et al., 2010).

The present study was performed to test the hypothesis (i) that there is a relationship between 2D:4D ratio and personality factors capable to be good predictors for choosing highly risky activities such as caving, in a sample of expert speleologists. Furthermore, we assessed (ii) whether 2D:4D ratio is related to their skills to regulate anxiety, emotions and mood state. Moreover, the purpose of this study was to evaluate (iii) the personological traits of expert speleologist and to study their regulation of anxiety, mood and emotions.

## Materials & Methods

### Participants

For this study, we have selected a sample of cavers belonging to sport associations located in Sicily. The sample of cavers consisted of 34 healthy participants, aged between 24 and 71 years (*M* = 39.70, *SD* = 9.81), including 18 males (*M* = 41.61, *SD* = 12.38) and 16 females (*M* = 37.56, *SD* = 5.40). Participants belonged to a group of expert cavers, who practice this sport from a period of a minimum of 24 months and a maximum of 540 months (*M* = 172.23, *SD* = 133.91), distributed as follows: 24 to 100 months (*N* = 14); 101 to 220 months (*N* = 12); from 221 to 400 months (*N* = 5); more than 400 months (*N* = 3). The frequency of annual caves explorations ranges from 2 to 50 (*M* = 23.23, *SD* = 14.56), distributed as follows: 2 to 10 descents (*N* = 9); 11 to 25 descents (*N* = 12); 26 to 40 descents (*N* = 9); more than 40 descents (*N* = 4).

Participants excluded from the study were those who: (i) had less of 24 consecutive months experience; and (ii) obtained a standardized score (T-score) ≥65 on the Lie Scale and at least on 3 major factors of the BFQ-2. The authors of the BFQ-2 indicate the latter criterion to identify the falsified personological profiles that should be excluded from data analysis.

The study obtained ethical permission from the University Enna Kore Internal Review Board for psychological research (13 January 2019). All participants were informed about the procedures of the study and the anonymity of their answers before providing their written consent to participate, in accordance with the Declaration of Helsinki.

### Digit Ratio measurement

The procedure for measuring the 2D:4D ratio provides the measurement of the digit length from the metacarpo-phalangeal crease to the fingertip (Coco et al., 2011; Massimino et al., 2019). The measure was performed by using a digital vernier callipers measuring to 0.01 mm and it was taken twice and then averaged. Ratios were calculated by dividing the length of the second digit by the fourth (2D:4D).

### Personality assessment

#### Big five questionnaire-2

The Big Five Questionnaire-2 (*BFQ-2*; Caprara et al., 2008). It’s a personality test based on the theory of big five that individuates five fundamental dimensions for the description and evaluation of personality. The constructive validity was confirmed by the relationship with other tools proposed for the measurement of personality, including the neo-personality inventory (NEO-PI, Costa & McCrae, 1990; Costa & McCrae, 1992). The test is structured in 134 items, using a 5-point Likert scale, ranging from “Absolutely false for me” to “Absolutely true for me”.

The Big Five Questionnaire-2 assesses personality traits divided into 5 major factors, each of which divided into two sub-dimensions:

• *Extraversion (outgoing/energetic vs. solitary/reserved):* energy, surgency, optimistic emotions, assertiveness, social openness, talkativeness and the propensity to seek inspiration with other people. In BFQ-2, it is called Energy and it is divided into two sub-dimensions: *Dynamism* and *Dominance.*
• *Agreeableness (friendly/compassionate vs. analytical/detached):* a propensity to be empathetic and supportive towards others; it measures whether a person is generally well tempered or not; it also provides an index of one’s trustful and unselfish nature. In BFQ-2, it is called Friendliness and is divided into two sub-dimensions: *Cooperativeness* and *Politeness.*
• *Conscientiousness (efficient/organized vs. easy-going/careless):* propensity to be organized and responsible, to demonstrate self-discipline, to act obediently, to aim for success, and to choose planned conduct. In BFQ-2, it is divided into two sub-dimensions: *Scrupulousness* and *Perseverance.*
• *Neuroticism (sensitive/nervous vs. secure/confident):* the attitude to experience unpleasant emotions effortlessly, such as irritation, nervousness, depression, and susceptibility. It also indicates the grade of emotive solidity and impulse control. In BFQ-2, it is called Emotional Stability and it is divided into two sub-dimension: *Emotion control* and *Impulse control.*
• *Openness to experience (inventive/curious vs. consistent/cautious):* it reveals the grade of intelligent interest, creativeness and an inclination for novelty and variability. In BFQ-2, it is called Openness and is divided into two sub-dimensions: *Openness to culture* and *Openness to experience.*
• A sixth factor, representing a control scale, labelled *Lie Scale,* consisting of two sub-dimensions *(Lie egoistic* and *Lie moralistic),* was added. This scale evaluates the participant’s tendency to provide a false profile of him/herself. The items are rated on a 5-point Likert scale ranging from 1 (absolutely false) to 5 (absolutely true).

Costa & McCrae (1992) report for BFQ-2 an internal consistency with Cronbach alphas ranging from .73 to .86. The coefficients alpha for the Italian questionnaire are also very high (Caprara et al., 2008). These authors also found an alpha of .74 for the Lie Scale.

### Risk taking inventory

The Risk Taking Inventory (*RTI;* Woodman et al., 2013). This tool measures the risk-taking behavior of a person who is inclined to perform a high-risk sport. The RTI consists of seven items, clustered in two factors: *Deliberate Risk Taking, DRT* (e.g., He/she actively seeks out dangerous situations) and *Precautionary Behaviors, PB* (e.g., He/she takes time to check for potential hazards). Items are classified on a 5-point Likert scale (1 = never; 5 = always).

### Mood measurement

#### Profile of mood states

The Profile of Mood States (*POMS;* McNair, Lorr & Droppleman, 1971; Italian adaptation by Farné et al., 1991). It provides a measure of mood states. The respondents must complete the POMS questionnaire by rating each item on a 5-point Likert scale with anchors reaching between ‘Not at all’ to ‘Extremely’. Internal consistency is particularly high ( *r* = 0.90). The items are combined to form six separate subscales: *Tension-anxiety (T), Depression-dejection (D), Anger-hostility (A), Vigor-activity (V), Fatigue-inertia (F) and Confusion-bewilderment (C).* The 6 subscale T-scores were then be combined to form an overall index of affectivity that is known as *Total Mood Disturbance (TMD* =* T* +* D* +* A* −* V* +* F* +* C).*

#### State-trait anxiety inventory form Y

The State-Trait Anxiety Inventory Form Y (*STAI-Y*; Spielberger et al., 1983; Italian adaptation by Pedrabissi & Santinello, 1997). It is a psychological test based on a 4-point Likert scale and consists of 40 items on a self-report basis. The STAI-Y assesses two types of anxiety—*State Anxiety,* or anxiety about an event, and *Trait Anxiety,* or anxiety level as personological trait. Higher scores are positively related to higher levels of anxiety.

### Procedure

The participants were examined, in a quiet room, in meetings lasting about 25 min. One of the authors (S.M.) photocopied the right hands of the participants to determine 2D:4D ratio, which appears to be more sensitive to prenatal androgens around the 9th week of gestation and it is one of the primary creases of the hand (Manning, Churchill & Peters, 2007; Williams et al., 2000; Kimura et al., 1990). The 2D:4D ratio was measured and psychological assessment tests were used, such as BFQ-2, POMS, STAI-Y and RTI, at about 2 h prior the new cave exploration.

### Data analysis

Data were collected and averaged; multiple linear regression and the correlation coefficient of Pearson were also calculated. Significance was set at *p* < 0.05. All descriptive statistics are described as mean  ± SD. Statistical analyses were performed using the SPSS v. 25.

## Results

Univariate analyses of 2D:4D ratios of the 34 cavers, aged between 24 and 71 years (mean, *m* = 39.70;  ±9.81 SD), including 18 males (*m* = 41.61;  ±12.38 SD) and 16 females (*m* = 37.56;  ±5.40 SD), provides values distributed between 0,864 and 1,087, with a mean value of 0,969 (±0,053 SD) and it shows no significant difference between men (0,957  ± 0,045 SD) and women (0,985  ± 0,057 SD). No significant correlations between 2D:4D and cavers affective states (STAI-Y, POMS) were found.

**Figure 1:**
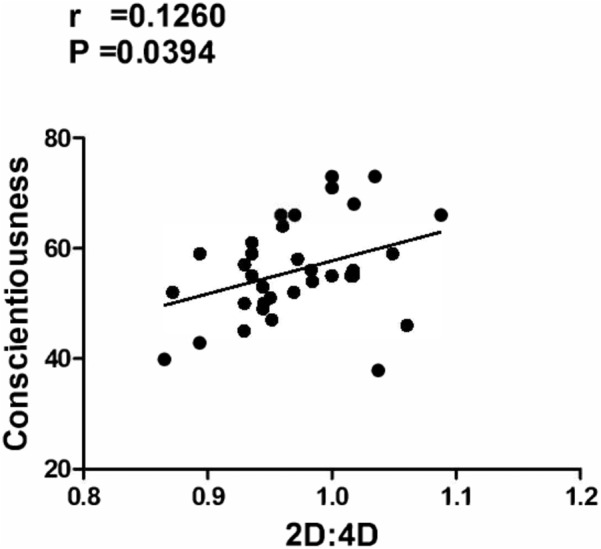
Significant correlation between BFQ-2 and 2D:4D Positive correlation between Conscientiousness (BFQ-2) and 2D:4D (second-to-fourth digit ratio). Significant correlation between BFQ-2 and 2D:4D.

Figure 1 illustrates the significant correlations between 2D:4D and the BFQ-2 factors. Data analysis shows a significant positive correlation between 2D:4D and the factor Conscientiousness (*p* = .0394; *r* = 0.1260) of BFQ-2.

**Figure 2:**
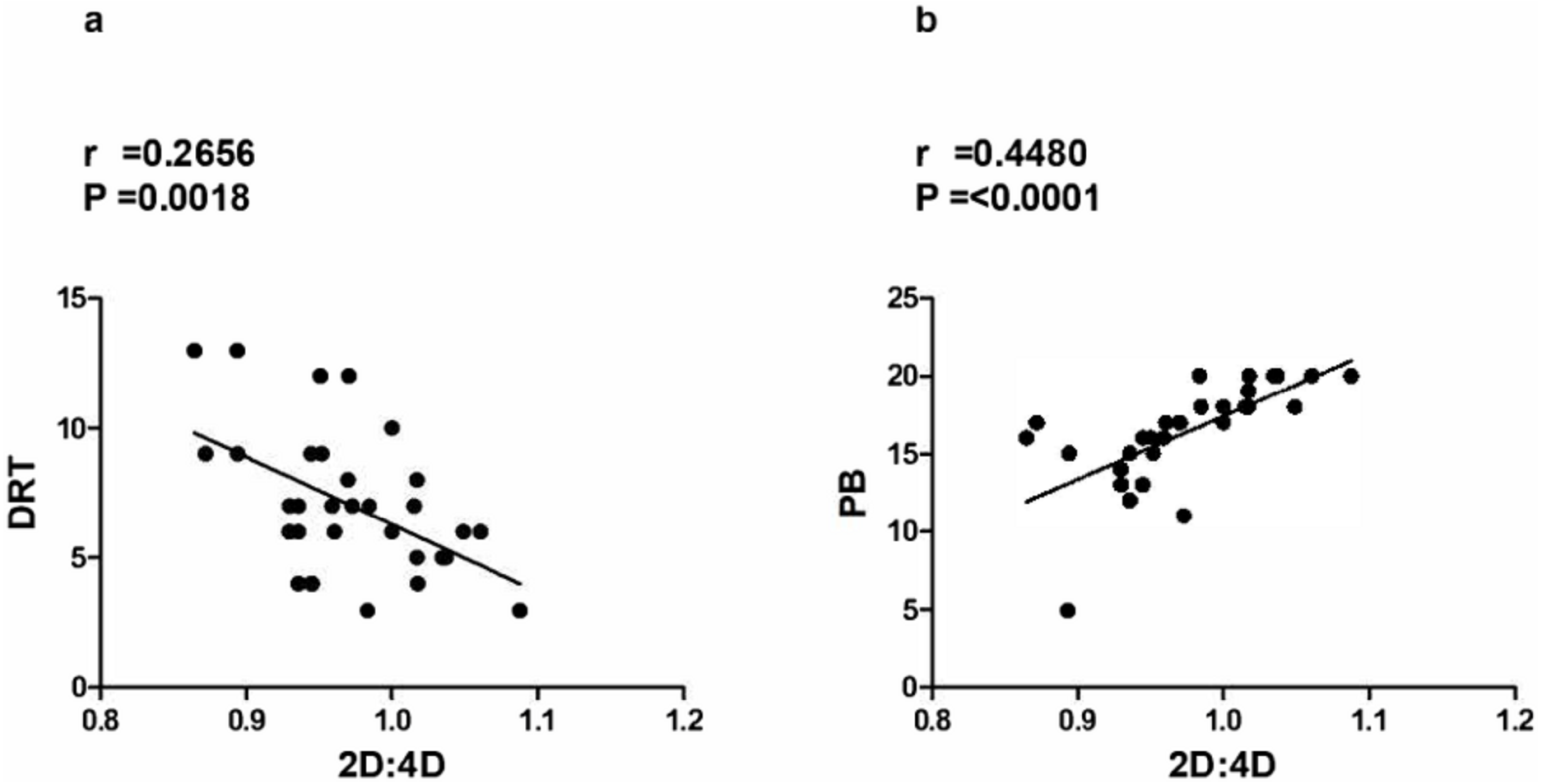
(A) Negative correlation between DRT (Deliberate Risk Taking, RTI) and 2D:4D (secondto- fourth digit ratio); (B) Positive correlation between PB (Precautionary Behavior, RTI) and 2D:4D (second-to-fourth digit ratio). Significant correlation between RTI and 2D:4D.

Furthermore, as illustrated in Fig. 2, it is interesting to note the correlations between 2D:4D and RTI factors (Deliberate Risk Taking, or DRT, and Precautionary Behavior, or PB). As can be seen in Fig. 2A, it was emerged a significant negative correlation between 2D:4D and DRT ( *p* = .0018; *r* = 0.2656) and, on the other hand, in Fig. 2B, a significant positive correlation between 2D:4D and PB (*p* < .0001; *r* = 0.4480).

Mean values (±SD) of the five major factors, the sub-dimensions and the Lie Scale of BFQ-2 are shown in Table 1 below.

**Table 1:** Means and Standard Deviations of BFQ-2 5.

	*Means*	±* SD*
***BFQ-2 5 FACTORS***		
Energy	51.20	9.58
Agreeableness	54.58	8.70
Coscientiousness	55.94	8.89
Emotional Stability	54.55	10.51
Openess	61.88	7.16
Lie Scale	54.70	8.52
***BFQ-2 SUB-DIMENSIONS***		
Dynamism	54.67	9.12
Dominance	48.50	10.31
Cooperativeness	56.64	8.52
Politeness	51.88	8.51
Scrupulousness	54.00	10.23
Perseverance	56.79	8.36
Emotion Control	54.32	9.80
Impulse Control	54.29	10.65
Openess to Culture	59.67	6.82
Openess to Experience	60.05	7.17
Lie Egoistic	55.35	7.85
Lie Moralistic	53.91	9.46

**Notes.**

Normative Reference: scores very low = 25–35; Low scores = 35–45; Scores normal = 45–55; High score = 55–65; Very high scores = 65–75 (Caprara et al., 2008).

As can be seen in the Table 1, Openess has the highest mean score (61.88; ± 7.16 SD), while Energy has the lowest mean value (51.20; ± 9.58 SD). The sub-dimension Openness to Experience shows the highest mean value (60.05; ± 7.17 SD), whereas the sub-dimension Dominance reports the lowest mean score (48.50; ± 10.31 SD).

Table 2 shows the mean values (±SD) of the 6 factors of POMS and TMD index, as follows.

**Table 2:** Means and Standard Deviations of POMS factors and TMD index.

*Poms factors*	*Means in T-scores*	*Means in raw-scores*	±*SD in t-scores*	±*SD in raw-scores*	*Range*
Tension	43.79	5.76	4.50	2.69	0–36
Depression	45.23	4.00	5.39	4.87	0–60
Anger	46.79	5.08	7.26	5.40	0–48
Vigor	57.70	19.47	7.01	4.35	0–32
Fatigue	46.82	4.55	7.35	3.29	0–28
Confusion	47.55	7.00	6.14	2.77	0–28
TMD	172.50	–	26.02	–	0–200

**Notes.**

TMD Total Mood Disturbance (TMD = T + D + A − V + S + C)

Normative Reference (expressed in raw-scores): Males means: T = 12.9; D = 13.1; A = 10.1; V = 15.6; F = 10.4; C = 10.2. Female means: T = 4 13.9; D = 13.8; A = 9.3; V = 15.6; F = 10.7; C = 11.7 (Farné, Sebellico, Gnugnoli & Corallo, 1991)

As illustrates, the factor Vigor has the highest mean score (57.70; ±7.01 SD), whereas the factor Tension has the lowest mean value (43.79; ±4.50 SD). Furthermore, the table shows the mean values of TMD index (172.50; ±26.02 SD); the TMD is calculated by the sum of factors, subtracting the value of the factor Vigor (TMD = T + D + A − V + S + C), that is the only factor in negative relationship with the other five factors.

Moreover, Table 3 shows the mean values (±SD) of Anxiety State and Trait Anxiety of STAI-Y.

**Table 3:** Means e Standard Deviations of State-Trait Anxiety (STAI-Y).

*STAI-Y*	*Means*	*SD*
State Anxiety	48.52	5.85
Trait Anxiety	49.41	6.76

**Notes.**

Normative Reference: Range min–max = 20–80; State-Anxiety males: m = 36.00; SD = 9.70; State-Anxiety female: m = 39.93; SD = 11.00. Trait-Anxiety males: m = 36.47; SD = 9.60; Trait-Anxiety female: m = 41.27; SD = 9.68 (Pedrabissi & Santinello, 1997)

The Trait Anxiety shows the higher mean value (49.41; ±6.76 SD) than Anxiety of State (48.52; ±5.85 SD).

**Figure 3:**
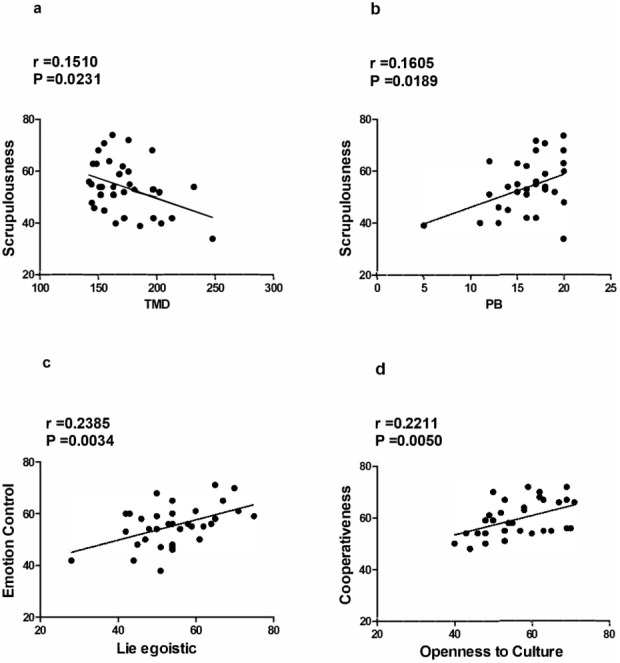
(A) Negative correlation between Scrupulousness and TMD (BFQ-2 and POMS); (B) Positive correlation between Scrupulousness and PB (BFQ-2 and RTI); (C) Positive correlation between Emotion Control and Lie Egoistic (BFQ-2); (D) Positive correlation between Cooperativeness and Openness to Culture (BFQ-2). Main statistically significant correlations.

Figure 3 shows the main statistically significant correlations between cavers personality factors (BFQ-2), their affective states (POMS, STAI-Y) and risk-taking behavior (RTI). Figure 3A shows a negative correlation (*p* = .0231; *r* = 0.1510) between the sub-dimension Scrupulousness and the index of TMD (BFQ-2 and POMS). Figure 3B shows a positive correlation (*p* = .0189; *r* = 0.1605) between the sub-dimension Scrupulousness and PB (BFQ-2 and RTI). Figure 3C shows a positive correlation (*p* = .0034; *r* = 0.2385) between sub-dimensions Emotion Control and Lie Egoistic (BFQ-2); Fig. 3D shows a positive correlation (*p* = .0050; *r* = 0.2211) between sub-dimensions Cooperativeness and Openness to Culture (BFQ-2).

Specifically, data analysis shows that Conscientiousness and its sub-dimension Scrupulousness (BFQ-2) are recurrent among significant correlations. In particular, the latter reports negative correlations with many factors of POMS, in addition to the TMD, as well as Depression (*p* = .0113; *r* = 0.1843), Anger (*p* = .0239; *r* = 0.1495), Fatigue (*p* = .0202; *r* = 0.1573), with the exception of Vigor with which it correlates positively (*p* = .0436; *r* = 0.1212). Finally, also Opennes to culture, (sub-dimension of Openness, BFQ-2), shows significant negative correlations with some POMS factors, such as Depression (*p* = .0182; *r* = 0.1623), Anger (*p* = .0209; *r* = 0.1558) and TMD (*p* = .0231; *r* = 0.1511).

## Discussion

The main goal of the present study was (i) to explore the relationship between 2D:4D ratio and personality factors capable to be good predictors for choosing highly risky activities, such as caving. Furthermore, our aim was (ii) to investigate whether 2D:4D ratio is related to skills of cavers to regulate anxiety, mood state and their emotions and (iii) to assess the personological and emotional features of this group.

As discussed, the brain development is influenced by prenatal androgens that would enhance its sensitivity to testosterone during life (Breedlove & Hampson, 2002; Tobet & Baum, 1987). Previous studies have proved that a longer fourth finger (i.e., lower 2D:4D ratio) is correlated to higher fetal androgen levels (Manning et al., 1998). Then, other studies (Berenbaum et al., 2009) asserted that, because of the remarkable within-group variability and between-group overlap, digit ratio is not considered a good marker to differentiate individual prenatal androgen exposition. Recently, Manning (2011) proposed that 2D:4D is definited not only by prenatal androgens but by the balance of prenatal androgen and prenatal estrogen during a limited period of fetal digit improvement. So, 2D:4D would be considered as a substitute marker for prenatal androgen exposition. Therefore, the 2D:4D ratio seems to be related to the individual personological features, as well as there is also increasing evidence that individuals with lower digit ratios, suggesting higher prenatal testosterone exposure, tend to report more general risk taking than who shows lower prenatal testosterone exposure (Kim, Kim & Kim, 2014; Barel, 2019).

In the sample of cavers analyzed in this study, the 2D:4D ratio appears related both with the propensity of Risk Taking (DRT) and, particularly, with the aptitude to assume a Precautionary Behavior (PB) in unsafe conditions (see Figs. 2A and 2B). Moreover, only Conscientiousness, a personological factor evaluated by the BFQ-2, was positively correlated with the 2D:4D (see Fig. 1). The relation between 2D:4D ratio and Conscientiousness, that is the disposition to have an organized rather than spontaneous behavior, could be considered as a significant predictor for a possible attitude for paying more attention in taking appropriate precautions during risky situations. Others studies (Nicholson et al., 2005) confirmed that the Emotional Stability, Agreeableness and Conscientiousness are negatively correlated with all domains of risk-taking, although we found a significant correlation only with the latter (Conscientiousness). In fact, the individuals with lower 2D:4D ratios seem to be less conscientious and scrupulous. This outcome is also coherent with the significant correlation between 2D:4D ratio and RTI factors (PB, DRT) (Figs. 2A and 2B). In fact, the correlations between 2D:4D and RTI factors are, respectively, positive with PB and negative with DRT, suggesting that there might be an early organizational effect of sex-steroids on some personality aspects reflecting the choice of the caving than other sports. Therefore, cavers with lower 2D:4D ratios seem to be less careful in taking precautions when they decide to take a risk. So, 2D:4D can be useful for predicting risk-taking behavior and some personological traits (Booth, Johnson & Granger, 1999; Austin et al., 2002; Fink et al., 2006; Apicella et al., 2008), such as Conscientiousness.

Furthermore, no significant association between 2D:4D ratio, POMS factors and STAI-Y factors was found. The lack of significant correlations would indicate that pre-natal androgen levels in cavers of our study would not seem to influence the affectivity state of the subjects, which would be regulated rather by the desire to bring off the common goal in the best possible way. In fact, this evidence seems to be provided by the negative correlation between the BFQ-2 factor Conscientiousness, in particular with its sub-dimension Scrupulousness, and the overall mood index, or TMD, provided by the POMS (which indicates a possible presence of disturbances of the mood by high scores), as Fig. 3A shows. In fact, the sub-dimension Scrupulousness, which by definition measures aspects concerning the caution, the methodical, order and attention to detail, is present in cavers in a consistent manner, and it correlates negatively also with the others factors of POMS. In this way, for cavers the success of their activity seems to be based on scrupulosity and an efficient affective control. This trend is present even though the single factors of POMS analyzed, whose values are much lower than the normative references, except, of course, for factor Vigor which has much higher values with respect to both the examined sample and the reference norms (see Table 2).

In addition, the Scrupulousness shows a positive correlation with the Precautionary Behavior (see Fig. 3B); this is would be related to the features of caving that implies responsibility and a careful behaviors to allow team collaboration and success of the group.

In particular, caves are a risky environment because of the high humidity, darkness and slippery conditions. Usually, explorations can continue for many hours and require extreme climbing and ropework (Pinna et al., 2017). Moreover, caving is a sport which assumes a team collaboration. In the caves cohesion and confidence between members of the cavers group is essential. In each team, everyone is responsible for the one who is behind him. Each caver should not control the one who stands before him, but the one who follows him. In this way, every action carried out by a member of the group influence the success of the whole group. This collaboration seems precisely aimed solely at this purpose. In fact, the data analysis does not show significant correlations regarding the BFQ-2 personality factor Agreeableness (i.e., the propensity to be empathetic and cooperative rather than suspicious and hostile towards others), nor with its sub-dimension Friendliness (i.e., whether a person is usually well moderated or not). Instead, a significant correlation was found only related to its sub-dimension Cooperativeness (i.e., willingness to work together with others), specifically with Openness to Culture, which evaluates the attitude to increase knowledge held. Therefore, it seems that the desire to explore hidden places and know new things is strictly linked to the cooperation with others as a way to achieve one’s goal (Fig. 3D). This correlations leads us to suppose that, in cavers, the group dynamics and interpersonal relationships within it are primarily related to the activities performed together, rather than simply “to making new friendships”, contrary to what is found for other high-risk sports, such as skydiving (Massimino et al., 2019).

Furthermore, among the factors of POMS, Tension is the factor with lowest mean value (see Table 2). As mentioned above, since caving is a type of activity that presupposes a responsibility to themselves and to others, an excessive level of tension would be not functional, probably, for the achievement of common result. Consistently, the correlation between the Lie Scale, in particular with the subscale Lie Egoistic (i.e., the tendency of the subject to appear brave and responsible in front of others), and Emotional Control (BFQ-2) brings out that, when the subject engages in activity, the implementation of personal qualities, as cooperating effectively with others or showing himself competent and brave, allows him to contain the anxiety and to regulate their emotional states (see Fig. 3C). This too seems to be functional to the kind of activities they perform (Elias & Dunning, 1986).

Moreover, this finding seems to be confirmed by the relationship between personality factors and mood, in which the sub-dimension Lie Egoistic of BFQ-2 correlates negatively with the Total Mood Disturbance index (TMD). Therefore, as described by Barlow, Woodman & Hardy (2013), emotion regulation emerges as an important feature in the high-risk sport, as well as caving.

Finally, the already mentioned BFQ-2 factor Openness (i.e., the degree of intellectual curiosity), in particular its sub-dimensions Openness to Culture (i.e., willingness to acquire new knowledge) and Openness to Experience (i.e., disposition to live new experiences), seems to play an important role in our sample, showing the higher mean values in this dimension than other personality factors (see Table 1). In addition, the factor Openness to Culture correlates negatively with some factors of POMS, such as Depression, Anger and TMD, and positively with some sub-dimensions of personality, such as Dominance, Scrupulousness and, as discussed above, Cooperativeness. This allows us to make some reflections on the importance of the factor Openness related to people who practice this kind of high-risk sport. In particular, the Openness to Culture (described in terms of tendency of the subject to increase their knowledge), rather than the Openness to Experience, seems to be a predominant feature in the cavers, as partially hightlight above. This feature could be considered “predictive” in the choice of an activity, such as caving, which requires curiosity, perseverance and a great planning of cave exploration. So, by the examination of the biographic characteristics of the sample of cavers, it could be observe that it is constituted by 62% university graduates and by 35%, at least, high-school graduates, placing them in a medium-high cultural level. It is possible, therefore, that the cultural level could be a factor that influences or reflects the choice of this kind of high-risk activity.

## Conclusions

In summary, our study showed that 2D:4D is confirmed as an reliable index able to predict risk-taking behaviors, as well as the Consciousness factor, in particular for predicting the precautionary behaviors in unsafe conditions. In addition, all participants seem to have a good attitude to collaboration, in terms of goal-directed strategy and an adequate management of negative affective states, useful to maintaining a good level of stress within the group. Finally, the choice of this type of high-risk sport, differently from offhand activities such as skydiving, could reflect the cultural level of our sample. However, the present study has some limitations due to the size of the sample. This fact is due to the selection of the participants, as we have considered only groups that practice this sport in Sicily. Moreover, increasing the sample and involving groups from other countries, it would provide a more homogeneous sample about age span. Another limitation is the lack of assessment of women and men separately.

The future goal of this project should be to examine other types of high-risk sport activities and also to compare them with non-sporting subjects (as control group) in order to highlight analogies and differences between groups.

##  Supplemental Information

10.7717/peerj.8029/supp-1 Data S1 Variables of all scales

## Additional Information and Declarations

### Data Availability

The following information was supplied regarding data availability: The raw data are available as a Supplemental File.
